# Can QT dispersion improve the accuracy of stress ECG TMT in detecting myocardial ischemia in chronic stable CAD patients? A stress myocardial perfusion imaging study

**DOI:** 10.1186/s43044-020-00126-5

**Published:** 2021-01-07

**Authors:** Mohammad Abdalla Eltahlawi, Ahmed Mohamed Sanad, Kamel Hasan Ghazal, Ahmed Taha Abdelwahed

**Affiliations:** 1 Zagazig University, Zagazig, Egypt; 2 Kobri El-Kobba Military Hospital, Cairo, Egypt

**Keywords:** Stress imaging, QT dispersion, QT dispersion difference, Chronic coronary syndrome, Nuclear scan, Ischemia

## Abstract

**Background:**

QT dispersion (QTd) is related to regional variations in myocardial repolarization. Our study aims to assess the value of QTd in prediction of myocardial ischemia and its severity during stress imaging.

We enrolled one hundred patients having stable coronary artery disease (CAD) and fulfilling the “Appropriateness criteria for cardiac radionuclide imaging” (MPI). They were divided into group I including patients with MPI-detected ischemia (50 patients) and group II including patients with normal perfusion scan (50 patients). We excluded unstable CAD and all other causes affecting QTd. During isotope scan, ECGs were taken and QTd was calculated at rest and at maximum heart rate.

**Results:**

QTd was significantly higher in the ischemic group both at rest and exercise (*P* = 0.000). QTd difference, the difference between QTd at rest and stress, was calculated. QTd difference was significantly lower in normal than in ischemic group (*P* = 0.003). There was a significant positive correlation between QTd difference and defect size (*P* = 0.04).

**Conclusion:**

QTd increases in ischemia and the QTd difference (between rest and stress) correlates positively with severity of ischemia.

QTd and QTd difference could be used to improve the accuracy of stress imaging test.

## Background

Different methods have been introduced and used for diagnosing CAD and investigating the need for revascularization such as the exercise tolerance test (ETT) and imaging techniques, e.g., SPECT (single-photon emission computed tomography).

Although the results obtained by SPECT can provide important and reliable information about patients’ prognosis, this method also has some disadvantages including exposure to ionizing radiation and controversial reports about its degree of accuracy [1].

In 1990, Day CP et al. [2] coined the term QT dispersion and concluded that it was related to regional variations in myocardial repolarization in ECG.

Clinical and experimental data have shown that QT interval prolongation measured from the standard 12-lead ECG is a risk factor for ventricular arrhythmia and sudden death in patients with and those without previous acute myocardial infarction (AMI). The Rotterdam QT project [3] showed that QT prolongation is a risk factor for sudden death independent of age, history of AMI, heart rate, and drug use. There is further strong support for the hypothesis that interlead variations in QT interval reflect regional variations in ventricular repolarization, and increased dispersion of ventricular recovery time is believed to provide a substrate that supports serious ventricular arrhythmias [4].

Some researchers have suggested to use QT interval (QTI) changes in response to exercise-induced ischemia instead of ST-segment changes in order to increase the diagnostic accuracy of ECG. The underlying reason for using QTI for diagnosing ischemia is that exercise-induced ischemia causes heterogeneity in the ventricular repolarization. QT dispersion (QTd) is one of the QTI parameters used more frequently than the other parameters [5].

We aim to investigate the correlation between QTd, during rest and maximum exercise ECG, and the severity and extent of myocardial ischemia detected by myocardial perfusion imaging technique.

## Methods

Patients referred to our center (Heart Specialized Hospital of Kobri El-Kobba Military Medical Complex) between July 2018 to December 2018 were enrolled.

### Type of the study

Case—control study.

### Inclusion criteria

We included patients having symptoms suggesting chronic coronary syndrome (CCS) and subjected to a clinically indicated myocardial perfusion imaging for the evaluation of the coronary arteries. All included patients have fulfilled the “Appropriateness criteria for cardiac radionuclide imaging.”

### Exclusion criteria

Patients with (1) previously known congenital heart disease, (2) unstable angina, (3) severe valvular heart disease, (4) chronic renal failure on regular hemodialysis, (5) inter- or intraventricular conduction disturbance (IVCD) in the ECGs including RBBB and LBBB, (6) non-sinus rhythm including atrial fibrillation or implantable pacemaker rhythm, (7) patients taking medications that affect QT interval (e.g., macrolide antibiotics, amiodarone, sotalol).

All patients were subjected to complete history taking with special emphasis on age, sex, and risk factors (including hypertension, diabetes mellitus, smoking, renal impairment, hepatic affection, family history suggesting any cardiac problem) in addition to general and local cardiac examination.

All patients underwent the following investigations: 12-lead ECG, transthoracic echocardiography, treadmill exercise stress ECG, and SPECT imaging.

During isotope scan, ECGs were serially taken in different stages; therefore, ECGs taken at the beginning of the scan were used to calculate QTd at rest and those taken at the time of maximum heart rate for calculating QTd under stress.

For QT dispersion (QTd) calculation, the difference between the minimum and the maximum QT interval on the 12 lead ECG was used if at least eight leads were suitable for analysis [6]. The ECG was performed with a frequency of 50 HzAC and velocity of 25 mm/s. ECG analysis and QTd calculations were done manually using the EP calipers program (electronic android phone or tablet program, more accurate, easier to use, and less mechanical errors).

A simultaneous 12 lead ECG, taken on the day of the stress study of MPI, was used for QT measurements. The QT interval was taken as the interval from the onset of the QRS complex to the end of the T wave, defined as the intersection of the isoelectric line and the T wave. In the presence of a U wave, the end of the QT interval was taken to be the nadir between the T and U wave peaks. No extrasystolic or post extrasystolic QT intervals were included. The maximum QT interval was corrected for heart rate using the Hodges formula (QTc = QT + 0.00175(HR – 60)).

QTd difference, which is the difference between QTd at rest and at exercise (stress), was also calculated.

All scans were performed using SPECT (Gamma camera Siemens Symbia-E series). The studies were performed according to established guidelines and institutional protocols at the time of the scan. In order to calculate the number of segments involved in ischemic damage, we used the conventional 17-segment categorization. The summed ischemic score was also determined based on Cedars-Cinai calculations which show the total severity of ischemia and the number of damaged segments. The severity of ischemia in each segment was also determined based on the same calculations between zero to 4 (normal, mild, moderate, and severe ischemia as well as infarction). True myocardial perfusion defect was described with reference to (1) the defect size or extent (small, medium, and large), (2) severity of perfusion defect (mild, moderate, and severe), (3) extent of reversibility (reversible, irreversible, or reverse redistribution), and (4) localization (based on 17 segment model and coronary artery territory if possible).

Data were analyzed using computer software called R language (R-studio Version 0.99.484—© 2009-2015) to investigate the correlation between QTd and the scan results as well as to compare QTd values of different groups.

## Results

Ninety-six males and 4 females participated in our study (mean ± SD; age, 59.76 ± 9.1 years; range, 38-75) were divided into two groups depending on the perfusion scan results. Group I was the case group (50 patients, 50%) who had MPI-detected myocardial ischemia of different severity. Of patients suffering from myocardial ischemia, 23, 14, and 6 patients had mild, moderate, and severe ischemia respectively. While the remaining 7 patients had scarred myocardium. Whereas group II included patients with normal perfusion scan (50 patients, 50%) (Table 1).

**Table 1:** The demographic data and risk factors in both groups

Variables	Group 1		Group 2		***Ӽ***^**2**^ value	***P*** value
	Abnormal perfusion (no. = 50)		Normal perfusion (no. = 50)			
	No.	(%)	No.	(%)		
**Sex (male)**	49	98%	47	94%	0.2604	0.6098
**Hypertension**	33	66%	29	58%	0.382	0.5365
**DM**	22	44%	14	28%	2.1267	0.1447
**Smoking**	36	72%	33	66%	0.187	0.6654
**CABG:**	(9)	18%	(4)	8%	3.7874	0.2854
• 2 grafts	3	6%	0	0%		
• 3 grafts	5	10%	3	6%		
• 4 grafts	1	2%	1	2%		
**PCI:**	(20)	40%	(26)	52%	11.646	0.0703
• 1 stent	4	8%	14	28%		
• 2 stents	5	10%	7	14%		
• 3 stents	6	12%	5	10%		
• > 3 stents	5	10%	0	0%		
**MPI type:**						
• Normal	0	0%	50	100%		
• Ischemia	26	52%	0	0%		
• Scar	7	14%	0	0%		
• Mixed (same segment)	12	24%	0	0%		
• Ischemic/scar (different segments)	5	10%	0	0%		
**MPI severity degree:**						
• No ischemia	7	14%	50	100%		
• Mild	23	46%	0	0%		
• Moderate	14	28%	0	0%		
• Severe	6	12%	0	0%		

*CABG* coronary artery bypass graft, *DM* diabetes mellitus, *PCI* percutaneous coronary intervention

The mean (± SD) QTds at rest for patients with normal myocardium and those with myocardial ischemia detected by MPI were 15.7 (± 5.2) ms and 34.24 (± 13.91) ms [*P* = 0.0000] indicating high significant value.

QTd difference, which is the difference between QTd at rest and at exercise (stress), was compared between normal and ischemic groups. We found that the mean ± SD QTd was significantly lower in patients with normal perfusion scan (−5.82 ± 12.2 ms) than in those with abnormal myocardial scan (4.12 ± 19.08 ms) [*P* = 0.0033] indicating statistical significant value (Table 2).

**Table 2:** Comparison between both groups regarding QT parameters

Variables	Group 1		Group 2		***t*** value	***P*** value
	Ischemia		No ischemia			
	Mean	SD±	Mean	SD±		
**Age (years)**	60.6	8.640	58.92	9.56	−0.921	0.359
**QTd rest (ms)**	34.24	13.913	15.7	5.20	−8.826	0.000
**QTd exercise (ms)**	30.12	13.120	21.52	11.45	−3.493	0.001
**HR rest (bpm)**	82.14	15.389	79.56	12.64	−0.916	0.362
**HR exercise (bpm)**	135.84	16.146	137.4	16.23	0.481	0.631
**QTd difference**	4.12	19.809	−5.82	12.03	3.033	0.003
**EF% TTE (Simpson)**	52.66%	8.653%	62.14%	6.31%	6.259	0.000
**EF% MPI**	53.18%	6.921%	62.22%	4.14%	7.925	0.000

*bpm* beat per minute, *HR* heart rate, *SD* standard deviation, *ms* milliseconds

**Fig. 1:**
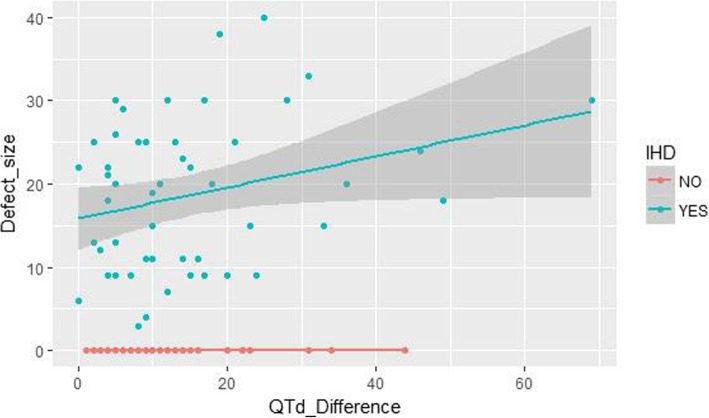
Correlation between defect size and QTd difference in both studied groups

Table 3 and Fig. 1 show a significant positive correlation between the defect size in MPI and QTd difference in group 1, (*r* = 0.279, *p* = 0.049).

**Table 3:** Correlation between QTd difference and defect size in patients with abnormal perfusion scan (group no. 1)

Variable	QTd difference	
	***r*** value	***P*** value
**Defect size**	0.2793	0.0495

When adding QTd difference to its result, the sensitivity, specificity, and accuracy of exercise stress ECG test was improved (Tables 4 and 5).

**Table 4:** Accuracy of stress ECG test in diagnosis of ischemia

Variable	Coefficient (β)	SE	***P*** value	Acc.	Sensitivity	Specificity
**Intercept**	−0.470	0.233	0.0434	0.68	0.6154	0.9091
**Stress ECG test**	2.773	0.777	0.0004			

*Acc* accuracy, *ECG* electrocardiogram, *SE* standard error

**Table 5:** Accuracy of stress ECG test to diagnose ischemia when adding the QTd difference

Variable	Coefficient (***β***)	SE	***P*** value	Acc.	Sensitivity	Specificity
**Intercept**	−1.082	0.378	0.0042	0.72	0.72	0.9191
**Stress ECG test**	2.863	0.789	0.0003			
**QTd difference**	0.048	0.023	0.0378			

### QT dispersion cut-off value to detect IHD patients

*N* = 47 patients only had > 0 change in QT-dispersion “exercise/rest” difference.

Table 6 represents novel results. Using cut-off point (9 ms), sensitivity reached 0.6, specificity 0.8, and AUC = 0.672. The sensitivity and specificity of QTd in the diagnosis and prediction of the ischemic events in MPI helped us to draw a frame for abnormal perfusion scan.

**Table 6:** Predictive value of QTd to detect IHD

Coefficient	Value	Confidence interval (CI) = 95%
**Cut-off**	9.000	
**Sensitivity**	0.600	0.406-0.773
**Specificity**	0.800	0.519-0.957
**AUC**	0.672	0.517-0.828
**Positive predictive value**	0.857	0.618-0.932
**Negative predictive value**	0.500	0.313-0.847

*AUC* area under the curve

**Fig. 2:**
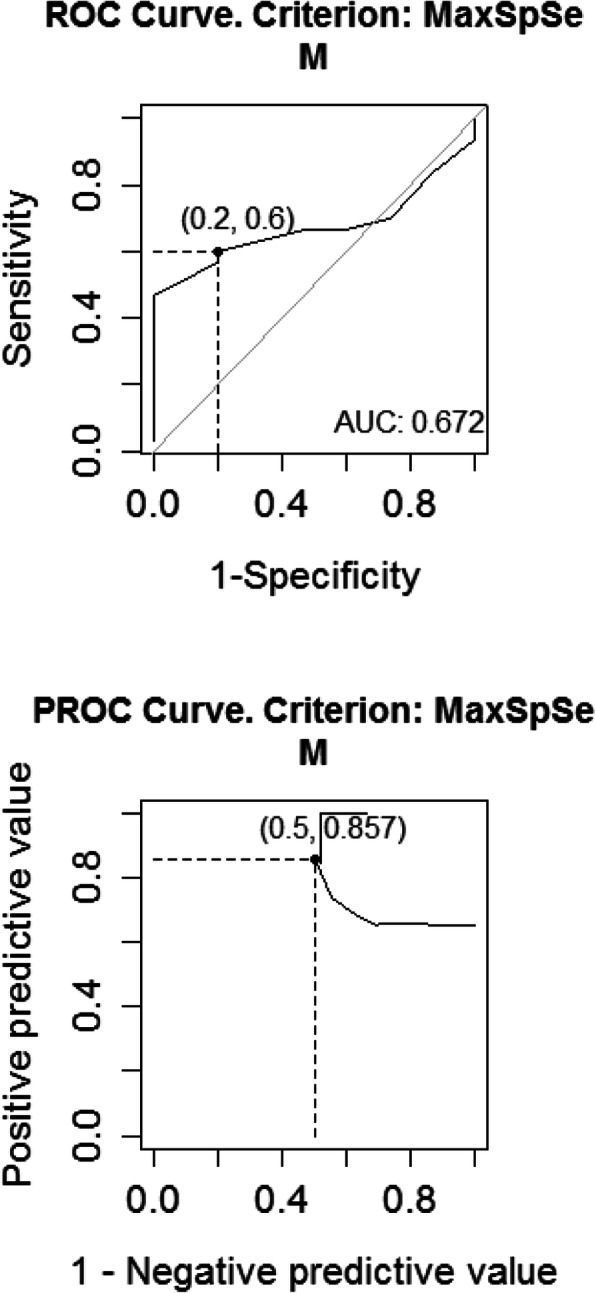
ROC curve of the sensitivity, specificity, and accuracy of QTd to detect IHD

Figure 2 represents the ROC curve of the sensitivity, specificity, and accuracy of QTd to detect IHD.

## Discussion

In our study, we examined the correlation between QTd and the severity and extent of myocardial ischemia detected by myocardial perfusion imaging. Our findings suggest that QTd values both at rest and had a significant relationship with the extent and severity of myocardial ischemia and rises with the increase in the severity and extent of ischemia.

A significant increase in QTd value using dipyridamole upon the induction of abnormal ventricular wall motion was previously reported which means that ischemia could change QTd value [7].

Okishige et al. [8] demonstrated that QTd increases reversibly during ischemia in patients with CAD. This increase in dispersion should be presumed to result from a combination of a local repolarization abnormality and altered conduction within ischemic areas.

Some researchers have not considered QTd as a standard criterion for assessing the homogeneity of ventricular repolarization. They stated that not only the accuracy of the standards relating to the dispersion but also the existence of a direct correlation between homogeneity of ventricular repolarization and QTd is in question [9].

In contrast, other researchers believe that QTd is a reliable measure for investigating abnormalities of myocardial repolarization and is able to predict severe arrhythmias after myocardial infarction and also mortality because of cardiovascular diseases [10]. This could be explained that the increase in the heterogeneity of ventricular repolarization in myocardial ischemia results in an increase in QTd value [11].

We compared the QTd value in patients who suffered from ischemia with normal people based on myocardial scanning and found that this parameter was identical in both groups at rest but significantly increased with the induction of stress in the ischemic group compared with the normal one.

Intra-group difference also exists so that the QTd value rises significantly with the increase in the severity of ischemia.

It was previously shown that, in patients without chest pain or ST segment depression during stress induction, QTd calculation immediately after stress induction can be very useful for diagnosing CAD [12].

Moreover, QTd increases to more than 60 ms under stress, with 70% sensitivity and 95% specificity that can be useful in the diagnosis of CAD [13].

The patients with CAD have longer corrected QT (QTc) intervals at peak heart rates during exercise. This finding provides insufficient evidence to support routine incorporation of QTc at peak heart rates into exercise test interpretation [14].

Teragawa and his colleagues also observed that stress induction using ATP infusion was associated with increased QTd only in patients with ischemia (ischemia and ischemia with scar), whereas in normal patients or those who suffered from scars, ischemia as the result of ATP infusion caused reduced QTd. They stated that in the normal group, ATP infusion may simultaneously cause a significant reduction of the maximum and minimum QT segment duration. QTd at baseline and during ATP infusion correlated with the ATP-SPECT imaging pattern. Therefore, the induction of ischemia was associated with an increase in QTd in the group with ischemic injuries, and a decrease in QTd in the group without ischemic injuries [15].

Randomized clinical trials (RCTs) have shown that the addition of coronary CT angiography [16, 17] or functional imaging [18] to stress ECG clarifies the diagnosis, enables the targeting of preventive therapies and interventions, and potentially reduces the risk of myocardial infarction compared with an exercise ECG [19].

In recent ESC 2019 Guidelines for the diagnosis and management of chronic coronary syndromes (CCS), exercise ECG alone has been downgraded as an alternative to diagnose obstructive CAD if imaging tests are not available (class II b, level of evidence b) [20], keeping in mind the risk of false-negative and false-positive test results [21, 22].

Regarding diagnosis of IHD, our study shows that the sensitivity and specificity of stress ECG test for detecting ischemia are 61% and 90% respectively. We noticed that addition of QTd values to the results of stress ECG improves the sensitivity to 72% with an irrelevant effect on the specificity (91%).

We found a strong relation between the QTd difference and the defect size in MPS in the ischemic group. The increase in the defect size is associated with increase in QTd difference.

Takase and colleagues also observed that QTd at rest in patients with simultaneous reversible and irreversible injuries and those with only irreversible injury was significantly higher than the normal group and the group with reversible injury. However, QTd under stress increased in the group with reversible injury but decreased in other groups [23].

It should be noted that the correlation between QTd and ischemic parameters in the scan is much lower at rest than under stress. As mentioned, such QTd increase under stress might be because of the increase in the heterogeneity of ventricular repolarization caused by the incorrect reaction of the ischemic myocardium to catecholamine or the abnormal flow of calcium ions [24, 25].

The findings of Schmidt and his colleagues [26] are not consistent with ours. In their study, they investigated the QTd value in the diagnosis of myocardial ischemia compared with myocardial scan using thallium 201 (Tl-201 SPECT). They observed that there was no significant correlation between QTd at rest and under stress and scanning parameters such as the degree of myocardial ischemia, the number of ischemic segments, and summed ischemic stress score. They gave many explanations such as differences in QT duration of up to 60 ms have been reported in individuals without structural heart disease and a significant range of overlap exists between healthy volunteers with QT dispersion of 30 ± 10 ms and patients with heart disease with 56 ± 23 ms. Another explanation may come from potentially gross inter- and intraobserver variability of QT dispersion assessment as reported elsewhere [26].

Masaki and his colleagues also stated that not QTd, but QT peak dispersion (QTpd) can be useful in detecting stress-induced myocardial ischemia [25].

In our study, we tested an index called QTd difference that actually shows the difference between QTd at rest and under stress. The comparison of this index between the normal and ischemic groups indicates that its value increases significantly in patients with myocardial ischemia. Since QT difference gives a resultant of the two values of stress and rest QTd, this can possibly be a more appropriate index for diagnosis of myocardial ischemia and the homogeneity rate of repolarization of ventricular cells [27]. Schmidt and colleagues also examined this factor in their study [26].

We found also that the cut-off for QTd in the diagnosis and prediction of the ischemia equaled 9 ms. We could conclude that under exercise-induced stress, patients who had negative stress ECG test for exercise-induced myocardial ischemia but their QTd value was more than (9 ms), those patients most probably had abnormal perfusion scan results.

The most eminent results of our study are those concerning with QTd difference and stress. When adding QTd difference to its result, the sensitivity, specificity, and accuracy of exercise stress ECG test was improved.

Considering the different results obtained from our research regarding the QTd value and based on the findings of this study, we can conclude that QTd can be clinically useful in identifying ventricular ischemic myocardial events.

Further studies are needed to determine diagnostic capabilities of QT dispersion, including its sensitivity and specificity which are currently under investigation to detect the extent that the QTd could be clinically useful in identifying ventricular ischemic myocardial injuries.

## Conclusion

QTd increases in ischemia and the QTd difference (between rest and stress) correlates positively with the severity of ischemia. QTd and QTd difference could be used to improve the accuracy of stress ECG treadmill test.

Patients who had negative stress ECG test for exercise induced myocardial ischemia with QTd difference value more than (9 ms) most probably have abnormal perfusion scan results. So, the QTd can be clinically useful in identifying ventricular ischemic myocardial events.

QTd represents a simple, quick, cheap, and resourceful tool to assist in study interpretation, clinical management, and therapy guidance.

### Limitations

1. Relative small number of sample size.
2. The study was conducted in a single center.
3. Most of the involved subjects were males.

## Data Availability

The data that support the findings of this study are available from the corresponding author upon reasonable request.

## Abbreviations

AMI: Acute myocardial infarction
CAD: Coronary artery disease
CCS: Chronic coronary syndrome
ECG: Electrocardiogram
EET: Exercise tolerance test
HR: Heart rate
IVCD: Interventricular conduction delay
LBBB: Left bundle branch block
MI: Myocardial infarction
MPI: Myocardial perfusion imaging
QTc: Corrected QT interval
QTd: QT dispersion
QTI: QT interval
RBBB: Right bundle branch block
RCTs: Randomized controlled trials
SPECT: Single-photon emission tomography
201Tl: Thallium-201
